# Silencing of the *MP* Gene via dsRNA Affects Root Development and Growth in the Invasive Weed *Mikania micrantha*

**DOI:** 10.3390/ijms252312678

**Published:** 2024-11-26

**Authors:** Zhenghui Ou, Yuantong Zhang, Qiang Wu, Kangkang Wang, Guangzhong Zhang, Xi Qiao, Ying Yan, Wanqiang Qian, Fanghao Wan, Bo Liu

**Affiliations:** 1 Shenzhen Branch, Guangdong Laboratory for Lingnan Modern Agriculture, Genome Analysis Laboratory of the Ministry of Agriculture and Rural Affairs, Agricultural Genomics Institute at Shenzhen, Chinese Academy of Agricultural Sciences, Shenzhen 518120, China18351212377@163.com (K.W.); qianwanqiang@caas.cn (W.Q.); 2 College of Plant Health and Medicine, Qingdao Agricultural University, Qingdao 266109, China; 3 State Key Laboratory of Crop Stress Adaptation and Improvement, School of Life Sciences, Henan University, Kaifeng 475004, China; 4 Shenzhen Research Institute of Henan University, Shenzhen 518000, China; 5 Department of Insect Biotechnology in Plant Protection, Institute for Insect Biotechnology, Justus-Liebig-University Giessen, Winchesterstraße 2, 35394 Giessen, Germany; ying.yan@agrar.uni-giessen.de

**Keywords:** *Mikania micrantha*, invasive weed, *MONOPTEROS* gene, root development, RNA interference, RNAi-based herbicides

## Abstract

*Mikania micrantha* (“mile-a-minute” weed) is a global invasive alien weed that can cause severe damage to agroforestry ecosystems and significant agricultural losses worldwide. Although chemical, manual, or mechanical control methods are widely used to control *M. micrantha*, RNA interference (RNAi)-based biocontrol methods have rarely been reported for this species. The *MONOPTEROS* (*MP*) gene, encoding an auxin response factor, plays an essential role in embryonic root initiation in *Arabidopsis thaliana*. In this study, we identified the *MP* gene from *M. micrantha* via orthologous gene analysis. A total of 37 *MP* orthologous genes was identified in 4 plants, including 9 *MP* candidate genes in *M. micrantha*, 13 in *Helianthus annuus*, 6 in *Chrysanthemum nankingense*, and 9 in *Lactuca sativa*. Phylogenetic analysis revealed that an *MP* candidate gene in *M. micrantha (Mm01G000655,* named *MmMP*) was clustered into one clade with the *MP* gene in *A. thaliana* (*AtMP*). In addition, both *MmMP* and *AtMP* contain a B3-DNA binding domain that is shared by transcription factors that regulate plant embryogenesis. To study gene function, dsRNA against *MmMP* (*dsMmMP*) was applied to the roots of *M. micrantha*. Compared with those of the controls, the expression of *MmMP* was reduced by 43.3%, 22.1%, and 26.2% on the first, third, and fifth days after *dsMmMP* treatment, respectively. The *dsMmMP*-treated plants presented several morphological defects, mostly in the roots. Compared with water-treated plants, the *dsMmMP*-treated plants presented reduced developmental parameters, including root length, number of adventitious roots, root fresh and dry weights, plant height, and aboveground biomass. Additionally, safety assessment suggested that this *dsMmMP* treatment did not silence *MP* genes from non-target plants, including rice and tomato; nor did it inhibit root growth in those species. Collectively, these results suggest that *MmMP* plays an important role in root development in *M. micrantha* and provides a potential target for the development of species-specific RNAi-based herbicides.

## 1. Introduction

*Mikania micrantha* is an extremely fast-growing invasive alien weed of the Asteraceae family that is native to Central and South America [1]. *M. micrantha* has the advantages of a strong ability to reproduce, fast asexual reproduction, and rapid nutritional growth. It is listed as one of the 100 most invasive species worldwide by the International Union for Conservation of Nature (IUCN) [2].and is also known as a “plant killer” [3,4]. *M. micrantha* is present in tropical Asia, parts of Papua New Guinea, islands in the Indian and Pacific Oceans, and Florida in the United States [4,5]. *M. micrantha* poses an immense threat to native plant species and wreaks havoc by climbing and winding around other plants to block their sunlight reception and photosynthesis, eventually resulting in the death of other plants [1,6]. As an invasive weed in cash crop fields and forests, the invasion of *M. micrantha* has caused economic losses of up to 4,000 hectares related to forest and crop production [7,8,9]. It also leads to a loss of genetic and species diversity, reduced soil and food web stability, and altered nutrient cycles [6].

Currently, the main control technologies for *M. micrantha* include chemical herbicide control, manual or mechanical eradication, and biological control [10,11,12]. Chemical herbicides are currently the most common control method for *M. micrantha*, given their fast effects and ease of operation. For example, glyphosate, paraquat, glufosinate, and 2,4-D have obvious control effects on *M. micrantha* [11,13]. However, studies have reported that the application of chemical herbicides to *M. micrantha* results in damage to surrounding plants, leading to reduced resistance of the native plant community to sub-invasions by *M. micrantha* [14,15]. On the other hand, manual or mechanical eradication can significantly reduce the coverage area of *M. micrantha* in a short period of time. However, this method is only effective in small areas and is not an effective means for large-scale control [16,17]. In recent years, researchers have focused on developing biological control technologies that use pathogenic bacteria and insects to control *M. micrantha*, such as *Actinote thalia pyrrha*, *Actinote anteas*, *Brevipalpus phoenicis*, and *Pachypeltis micranthus* [18,19]. These parasitic agents are not highly specific since they also harm other plants and are therefore not ideal for field applications [6,20].

RNA interference (RNAi) is a posttranscriptional gene-silencing technique [21]. The principle of RNAi is to induce the degradation of homologous gene sequences in organisms via the complementary pairing of double-stranded RNA (dsRNA) bases [22]. RNAi-based technologies show pronounced potential for use in agriculture, particularly for the management of destructive insect pests [23,24,25,26]. For example, a greater than 80% knockdown rate was observed by silencing the expression of the target gene *V-type proton ATPase subunit D* (*ATPD*) in the control of soybean aphids (*Aphis glycines*) [27]. There have been several relevant reports on the application of dsRNA to plant roots. Majidiani et al. evaluated the absorption of dsRNA by plant roots and found that long dsRNA molecules can also be absorbed and cause gene silencing in target insects that feed on leaves [27,28]. Konstantin et al. analyzed the importance of physiological conditions and different dsRNA application methods (brush spreading, spraying, infiltration, inoculation, needle injection, and pipetting) for the suppression of the neomycin phosphotransferase II (NPTII) transgene in *Arabidopsis thaliana* [29]. Jiang et al. applied a mixture of G2/dsSTM or G2/dsWER to roots, which led to a dramatic reduction in the expression of STM and WER in the entire seedling. The specific RNA interference of STM and WER mediated by G2-delivered dsRNA resulted in reduced SAM size and increased lateral roots, respectively [30]. Minsu et al. used dsRNA to treat *A. thaliana* by dipping and spraying, both of which can induce the inhibition of target gene expression after the application of target gene-specific exogenous dsRNA [31].

RNAi has been reported as a promising molecular control strategy, particularly in weed management. For example, the application of RNAi to manage glyphosate-resistant weeds has emerged as a new technology, providing a novel approach for weed control [32]. For the application of RNAi technology in *M. micrantha*, to our knowledge, only one study has reported the use of three types of RNAi molecules (double-stranded RNA, RNAi nanomicrosphere, and short hairpin RNA) to inhibit the gene expression of chlorophyll a/b-binding proteins in *M. micrantha*, resulting in yellow leaves and eventual wilt [33]. Previous studies have shown that *MP* (*MONOPTEROS*, *MP*), also known as auxin response factor 5 (*ARF5*), is an auxin response factor that plays a key role in embryonic root initiation in *A. thaliana* [34,35]. Mutants with complete loss of *MP* function die at the embryo stage due to their inability to form roots [36,37,38].

With the rapid development of molecular biology and the emergence of advanced sequencing technologies, the genome of *M. micrantha* has been sequenced and assembled [39]. On the basis of this genetic resource, in this study, we identified the *MP* gene from *M. micrantha* (*MmMP*). Owing to the biological characteristics of the rooting ability of each stem node in *M. micrantha*, we focused on developing an RNAi-based approach to knock down *MmMP* expression and therefore inhibit root development. The morphological phenotypes of the roots of *M. micrantha* were subsequently measured to evaluate the inhibition of growth. These findings revealed the functional role of *MmMP* in regulating root growth, making it a potential target for the development of RNAi-mediated herbicides.

## 2. Results

### 2.1. Identification of the MP Gene in M. micrantha

**Figure 1:**
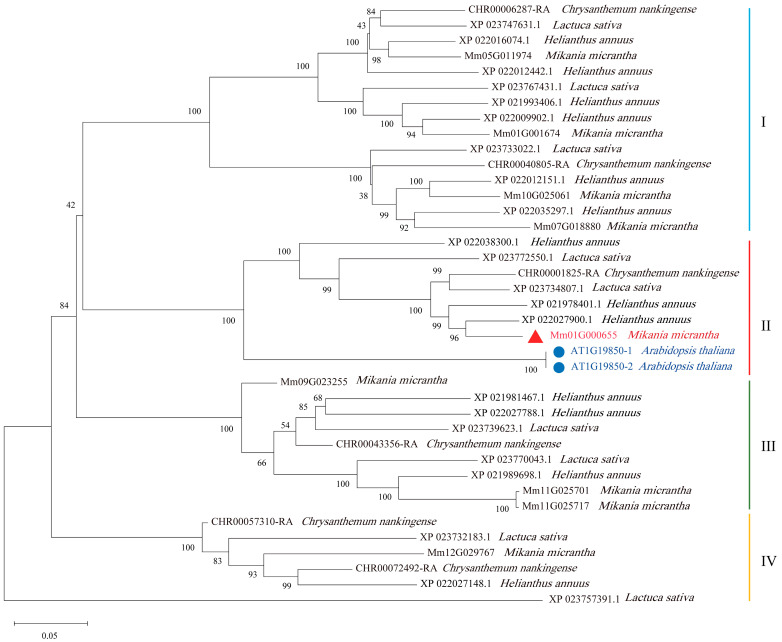
Phylogenetic analysis of *MP* genes from *M. micrantha* and four other species, namely, *A. thaliana*, *C. nankingense*, *H. annuus*, and *L. sativa*. I~IV represent the *MP* proteins of the five species divided into four independent clades. The tree was constructed via the neighbor-joining method based on the protein sequence alignments. The numbers on the branches represent bootstrap values obtained from 1000 replicates. The branch lengths are proportional to the percentage of sequence difference (scale bar, 0.05% difference). The red triangle represents the *M. micrantha* gene Mm01G000655; the blue circles represent two protein-encoding genes in *A. thaliana*.

To identify the orthologs of *MONOPTEROS* (*MP*) in the four plants, we used the amino acid sequence of *MP* (AT1G19850) in *A. thaliana* for alignment with the protein sequences of *M. micrantha*, *Chrysanthemum nankingense, Helianthus annuus*, and *Lactuca sativa*. A total of 37 *MP* orthologous genes (Supplementary Table S3), including 9 *MP* candidate genes in *M. micrantha,* 13 in *H. annuus*, 6 in *C. nankingense*, and 9 in *L. sativa*, were used to construct the phylogenetic tree (Figure 1). The results revealed that these proteins in these five species clustered into four clades (I, II, III, and IV). One gene (Mm01G000655) in *M. micrantha*, three genes (XP_0220279001, XP_021978401.1, and XP_0220383001) in *H. annuus*, one gene (CHR00001825-RA) in *C. nankingense*, and two genes (XP_023772550.1 and XP_023734807.1) in *L. sativa* were clustered in the same clade as two copies of *MP* in *A. thaliana* (named AT1G19850-1 and AT1G19850-2), suggesting that these orthologous genes may perform the same function (Figure 1).

**Figure 2:**
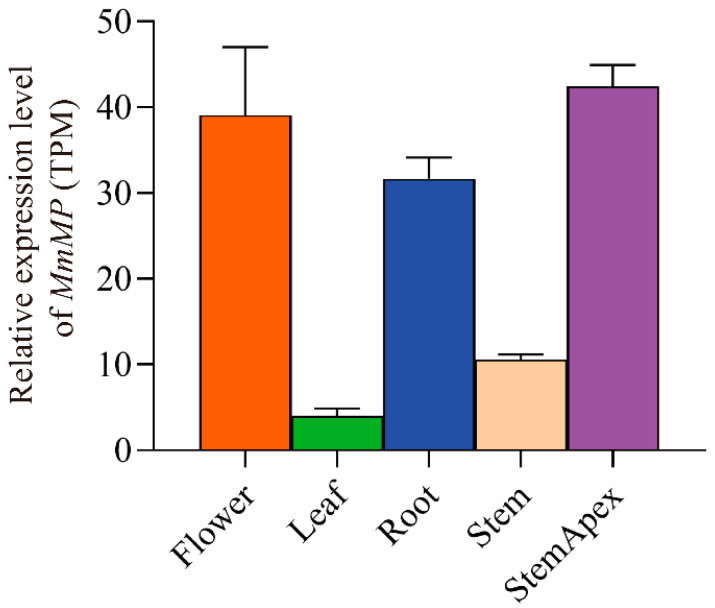
TPM values of candidate gene expression in different tissues. The values represent the means ± SE derived from five biological replicates.

In addition, the amino acid sequence of the Mm01G000655 gene was highly conserved with those of the *MP* genes (AT1G19850-1 and AT1G19850-2) in *A. thaliana* (Supplementary Figure S1 and Supplementary Table S4). Further domain analysis revealed that Mm01G000655 has typical auxin response factor (ARF) structural characteristics, i.e., one plant-specific B3-DNA binding domain, one auxin-encoding domain, and one AUX-IAA domain, which is consistent with the structure of the *MP* gene in *A. thaliana* (Supplementary Figure S1). Therefore, Mm01G000655 (named *MmMP*) of *M. micrantha* may perform the same function as *MP* in *A. thaliana*. Transcriptome analysis of different tissues revealed that the highest expression of *MmMP* was detected in the roots (TPM = 31.7), flowers (TPM = 39.1), and stem tips (TPM = 42.4), followed by the stems (TPM = 10.6) and finally the leaves (TPM = 4.0) (Supplementary Table S5). The expression level in the roots was 7.9 and 2.9 times greater than that in the leaves and stems, respectively (Figure 2).

### 2.2. Regulation of MP Gene Expression by dsRNA in M. micrantha Roots

**Figure 3:**
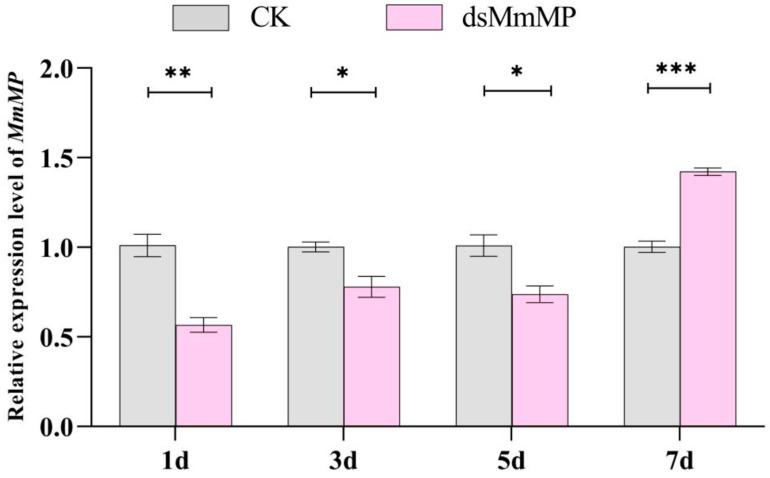
The abundance of *MmMP* transcripts was measured at different time points after the roots were soaked in water (CK) or *dsMmMP*. Each bar represents the mean ± SE derived from three biological replicates. A t-test was used to determine significant differences in plant-silencing efficiency in this study (* *p* < 0.05; ** *p* < 0.01; *** *p* < 0.001). CK indicates treatment with water, and *dsMmMP* indicates double-stranded RNA of the *M. micrantha MmMP* gene.

qRT–PCR was used to determine the effects of silencing via *dsMmMP*. Compared with that in the water-treated (CK) plants, *MmMP* expression in the *dsMmMP*-treated plants was inhibited by 43.3%, 22.1%, and 26.2% on the first (1 d), third (3 d), and fifth (5 d) days after dsRNA delivery, respectively (Figure 3). To further verify the inhibitory effect of dsRNA on the expression of target genes, we selected *dsEGFP* as a negative control. Compared with negative controls, *dsMmMP* significantly inhibited the expression of target genes, and the gene expression returned to normal levels by the seventh day (7d) (Supplementary Figure S2).

### 2.3. Effects of MmMP Gene Silencing on the Root Growth of M. micrantha

**Figure 4:**
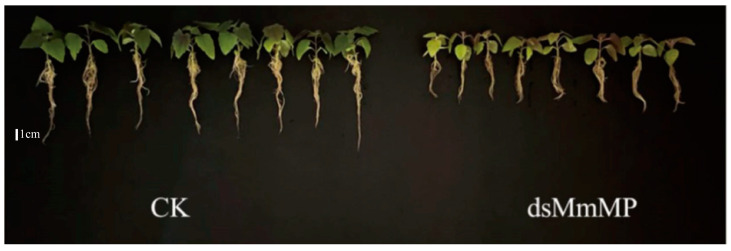
Effects of *dsMmMP* application on the phenotype of *M. micrantha*. Phenotypic changes in *M. micrantha* roots after continuous treatment for 15 days. CK, water control; *dsMmMP*, double-stranded RNA of the *M. micrantha MmMP* gene.

To determine whether *MmMP* gene silencing affects the growth and development of *M. micrantha*, the morphological phenotypes were observed after 15 days of treatment. Compared with the plants in the CK treatment, those in the *dsMmMP* treatment exhibited significantly inhibited root growth (Figure 4).

**Figure 5:**
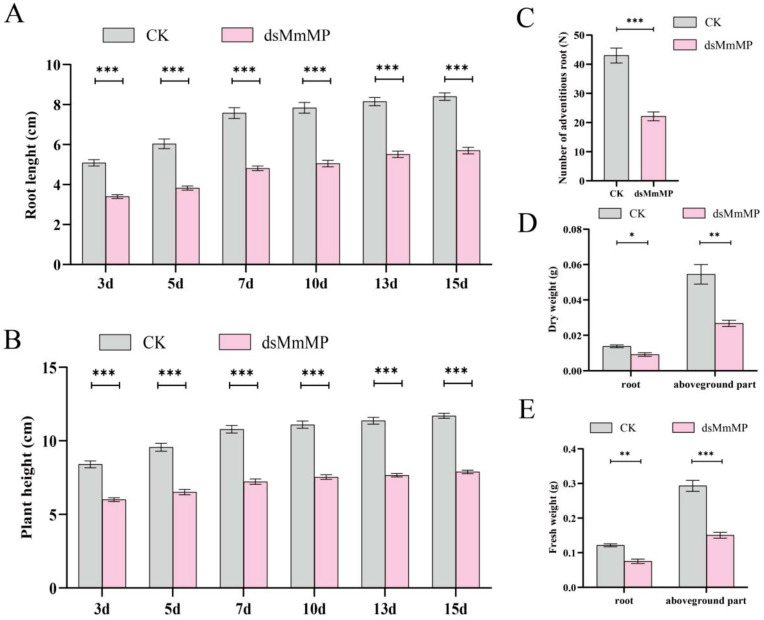
Statistical analysis of morphological indicators of *M. micrantha*. Statistical analysis of the length of the longest adventitious root (**A**), plant height (**B**), number of all adventitious roots (**C**), dry weights of all adventitious roots and aboveground parts (**D**), and fresh weights of all adventitious roots and fresh weights of the aboveground parts (**E**) after 15 d of continuous treatment. Each bar represents the mean and standard error for *n* = 8 biological replicates. A *t*-test was used to determine significant differences in plant-silencing efficiency in this study (* *p* < 0.05; ** *p* < 0.01; *** *p* < 0.001). CK, water control; *dsMmMP*, double-stranded RNA of the *M. micrantha MmMP* gene.

We subsequently measured the morphological phenotypes for 15 consecutive days under the dsRNA treatments. Compared with the CK plants, the plants treated with *dsMmMP* presented significantly shorter plant heights and lengths of the longest adventitious roots at the third, fifth, seventh, tenth, thirteenth, and fifteenth days. The average length of the longest adventitious root was inhibited by more than 30.1% under the *dsMmMP* treatment (Figure 5A). The average plant height was significantly inhibited by *dsMmMP*, representing an approximately 30.4% reduction (Figure 5B). After 15 days of treatment, the number of adventitious roots on average decreased by approximately 48.5% (Figure 5C). In addition, the fresh weights of the aboveground and belowground parts under the *dsMmMP* treatment significantly decreased by approximately 38.2% and 48.7%, respectively (Figure 5D). The average aboveground and belowground dry weights also decreased by 33.6% and 50.9%, respectively (Figure 5E).

### 2.4. Safety Assessment for DsMmMP

**Figure 6:**
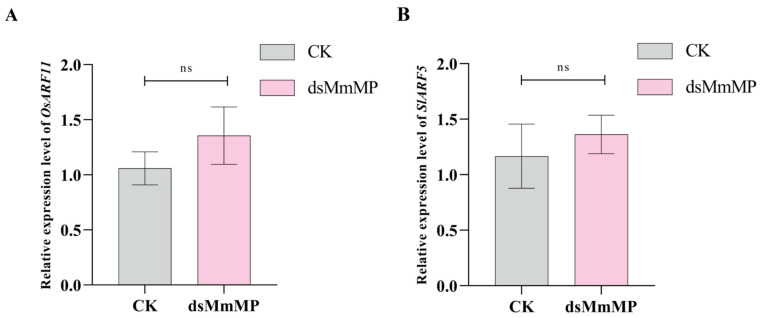
Effects of *dsMmMP* on *MP* gene expression in non-target plants. The expression of the *MP* gene from rice (*OsARF11*) or tomato (*SlARF5*) was measured 3 d after *dsMmMP* and water treatment. (**A**) Rice (**B**) Tomato Each bar chart represents the mean and standard error of *n* = 3 biological replicates, and the data were analyzed via one-way ANOVA to identify significant differences in plant-inhibition efficiency and multiple Tukey comparisons (*p* < 0.05). “ns” indicates no significant difference between the CK and *dsMmMP* treatments. CK, water control; *dsMmMP*, double-stranded RNA of the *M. micrantha MmMP* gene.

**Figure 7:**
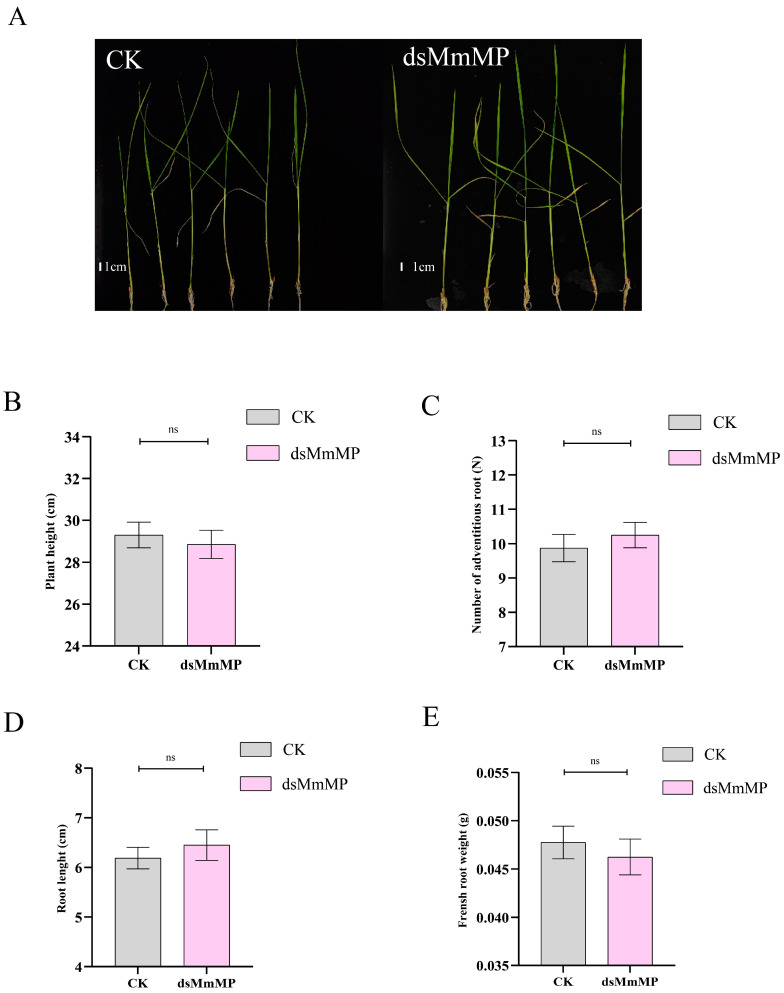
Effects of *dsMmMP* on the growth of *O. sativa*. (**A**) Image of a whole plant. (**B**) Plant height. (**C**) Number of adventitious roots. (**D**) Length of the longest adventitious root. (**E**) Fresh weight of all adventitious roots. Each bar represents the mean and standard error for *n* = 8 biological replicates, and the data were analyzed via one-way ANOVA to identify significant differences in plant-inhibition efficiency and multiple Tukey comparisons (*p* < 0.05). “ns” represents no significant difference between the CK and *dsMmMP* treatments. CK, water control; *dsMmMP*, double-stranded RNA of the *M. micrantha MmMP* gene.

**Figure 8:**
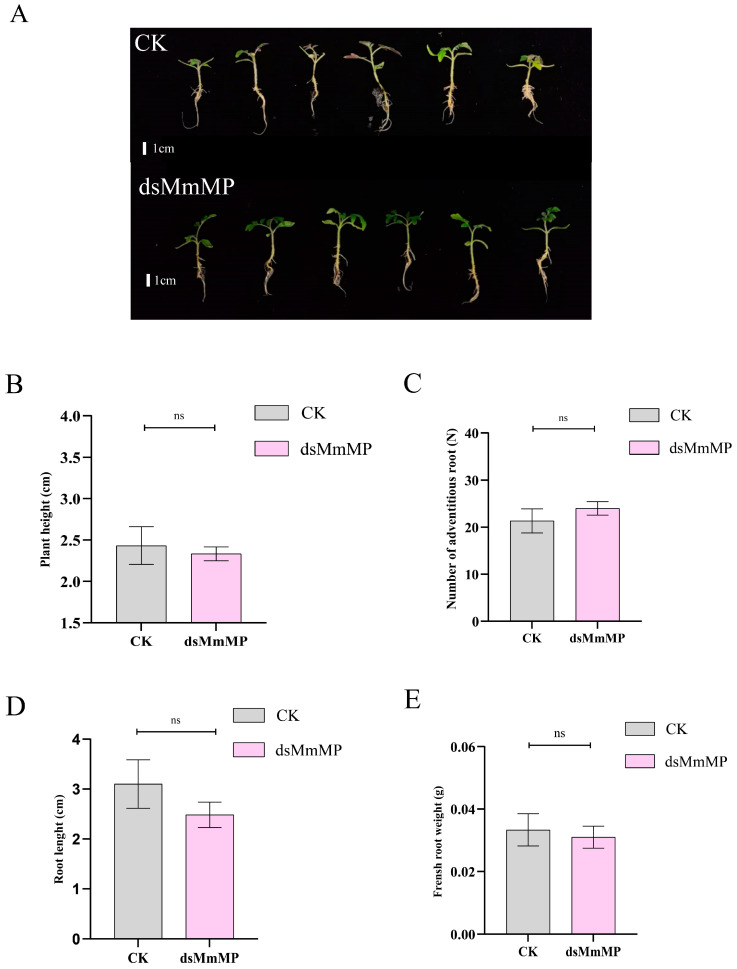
Effects of *dsMmMP* on the growth of *S. lycopersicum*. (**A**) Image of a whole plant. (**B**) Plant height. (**C**) Number of adventitious roots. (**D**) Length of the longest adventitious root. (**E**) Fresh weight of all adventitious roots. Each bar represents the mean and standard error for *n* = 6 biological replicates, and the data were analyzed via one-way ANOVA to identify significant differences in plant-inhibition efficiency and multiple Tukey comparisons (*p* < 0.05). “ns” indicates no significant difference between the CK and *dsMmMP* treatments. CK, water control; *dsMmMP*, double-stranded RNA of the *M. micrantha MmMP* gene.

To determine whether *dsMmMP* also targets the *MONOPTEROS* genes in rice (*OsARF11*) and tomato (*SlARF5*), we verified gene expression after *dsMmMP* and water treatment via qRT–PCR. The results revealed that the gene expression of *OsARF11* (Figure 6A) or *SlARF5* (Figure 6B) 3 d after *dsMmMP* treatment was not significantly different from that after-water treatment. This might be explained by the lower identity of *MmMP* to *OsARF11* (44.04%) and *SlARF5* (60.37%) at the amino acid level (Supplementary Figure S3). In addition, the nucleotide-binding region of *dsMmMP* also presented low identity compared with that of *OsARF11* (4.93%; Supplementary Figure S4A) or *SlARF5* (13.65%; Supplementary Figure S4B). Therefore, rice and tomato *MP* genes are not targeted by *dsMmMP*. To further investigate whether treatment with *dsMmMP* affects the root growth of rice and tomato, the morphological phenotypes were examined 15 days after treatment. The results suggested that the plant height, root length, number of adventitious roots, and fresh weight of roots of rice were not significantly different between the CK and *dsMmMP* treatments (Figure 7A–E). Moreover, these parameters in tomato did not significantly differ between the CK and *dsMmMP* treatments (Figure 8A–E). Consequently, *dsMmMP* does not seem to inhibit the root growth of rice or tomato.

## 3. Discussion

Sequence-specific knockdown of mRNA transcripts via RNAi provides countless opportunities for weed control. As a destructive invasive weed, *M. micrantha* has become a worldwide problem. Therefore, strong and effective biological strategies are urgently needed for the management of *M. micrantha*. RNAi is a conserved posttranscriptional gene-silencing process [40] that has been widely used in plant gene functional identification, quality improvement, and resistance research [41,42,43]. Previous studies have indicated that foliar application of dsRNAs encoding specific genes of plant pathogens triggered RNAi-mediated silencing of the target genes [43,44]. To data, the gene functional researches were major based on dsRNA-expressing transgenic plants and host-induced gene silencing [41,43]. However, few studies have reported the use of RNAi technology to control weeds. Recently, the importance of physiological conditions (plant age, time of day, soil moisture, high salinity, heat, and cold stresses) and different dsRNA application means (brush spreading, spraying, infiltration, inoculation, needle injection, and pipetting) for suppression of target gene were analyzed in *A. thaliana*, indicating that dsRNAs can suppress the expression of certain genes within an organism [29,45]. To select the gene that best suppresses *M. micrantha*, we chose a gene encoding an auxin-responsive protein, MP, as the target and designed the corresponding RNAi molecule. The use of RNAi technology to inhibit root development can effectively control the spread of *M. micrantha*, providing a theoretical basis for targeted control techniques that suppress the growth and development of *M. micrantha* through RNAi technology.

*M. micrantha* possesses strong reproductive capabilities, being able to reproduce sexually and spread through the production of a large number of seeds, as well as propagate through its strong asexual reproduction abilities. Therefore, the strong adventitious root formation at the nodes of *M. micrantha* stems is one of the main reasons for its ecological harm [1]. To improve the effectiveness of RNAi, it is particularly important to identify key target genes. This study focused on the inhibition of root growth and investigated the inhibitory effects of RNAi on root growth genes in *M. micrantha*, with the goal of biological control of this invasive weed. Previous studies have shown that the *MP *(*MONOPTEROS*, *MP*) gene is an auxin response factor that plays a key role in embryonic root initiation in *A. thaliana* [46,47]. Mutations resulting in complete loss of *MP* function result in embryo death due to the inability of *A. thaliana* to form root tips [38,48]. In this study, on the basis of genomics and transcriptome analysis, the *MmMP* gene was identified in *M. micrantha*. Further analysis revealed that the *MmMP* gene contained a conserved domain compared with the *MP* gene of *A. thaliana*, including a plant-specific B3-DNA binding domain, an auxin-resp, and an AUX-IAA [49,50]. We speculate that *MmMP* in *M. micrantha* has the same function as *MP* in *A. thaliana*. These findings reveal that the *MmMP* candidate gene may be important for further studies to inhibit the growth of *M. micrantha*.

At present, only a limited number of reports exist on the direct application of dsRNA to the exterior surface of plants, which can result in the silencing of endogenous target genes in the plant [51]. In this study, we analyzed the efficacy of target gene silencing in *M. micrantha* by soaking the roots of *M. micrantha* in dsRNA solution. The qRT–PCR results revealed that the expression levels of the *MmMP* gene were significantly reduced, indicating that RNAi was successful. Although the expression of the target gene was inhibited, the interference efficiency was not particularly high, which was speculated to be due to the relatively low concentration (0.3 μg/μL) applied, and increasing the dsRNA concentration could be considered in subsequent experiments. On the other hand, the relatively low efficiency might be because the applied dsRNA was not efficiently delivered inside the plant cell since it was taken up by the root and then transported through the xylem in woody plants [52]. To trigger intensive and global RNAi effects, they may need to be delivered inside the plant cell, either by mechanical damage to the cell wall [52] or through the use of nanoparticles [53] for efficient dsRNA delivery. Specifically, numerous studies have indicated that combining certain nanomaterials could significantly improve RNAi efficiency in some insects [54,55,56]. Moreover, studies have shown that, in plants, the application of dsRNA mixed with G2 nanoparticles to the root tip of *A. thaliana* markedly decreases the transcript levels of the target genes. Plants treated with G2/dsRNA exhibited slow growth, while dsRNA treatment alone did not lead to these effects [30]. In this study, we found that target gene expression in *M. micrantha* was significantly suppressed within 5 days after treatment, while the dsRNA may have lost its silencing effect on the seventh day. To improve the effects and duration of dsRNA action in plants, the use of nanoparticles may greatly facilitate the development of RNAi-based herbicides for *M. micrantha* and other weed pests.

Importantly, the results of the present study collectively revealed that *dsMmMP* had relatively strong and persistent inhibitory effects on plant development, verifying the gene function of *MmMP*. Phenotypic measurements revealed that the aboveground and belowground growth of *M. micrantha* was significantly inhibited after *MmMP* gene expression was reduced. Further safety assessment revealed that the nucleotide-binding region of *dsMmMP* shares low identity with those of *OsARF11* (4.93%) and *SlARF5* (13.65%), leading to be not significantly different between the water and *dsMmMP* treatments in rice and tomato, suggesting that the *dsMmMp* has an appropriate safety profile for important crops. On the other hand, the phylogenetic tree analysis of *MP* genes among Asteraceae plants, *A. thaliana*, rice, tomato, and *M*. *micrantha* indicated that the *MP* genes in *M. micrantha* are more distantly related to those in non-target plants such as rice and tomato (Supplementary Figure S5). Consequently, this further demonstrates the safety of the *dsMmMP* gene specific to *M. micrantha* against non-target plants. With respect to the study of root genes, our RNAi formulation can be applied through root irrigation, which is expected to make the application of RNAi biological herbicides possible, but the effectiveness of field control still needs to be further confirmed. Here, while we have demonstrated the safety of two non-target plants, the level of evidence may not be sufficient. In the future, we should continue to use a greater number of non-target plants to confirm the safety of off-target effects of dsRNA formulations and to verify the feasibility of using dsRNA-based herbicides to control invasive weeds. The development and use of RNAi bioherbicides can provide an efficient, safe, economical, and environmentally friendly method to effectively control *M. micrantha* in the field, which is highly important for advancing research on biological control techniques for *M. micrantha*.

## 4. Materials and Methods

### 4.1. Plant Material and Growth Conditions

*M. micrantha* seeds were collected from the farm of the Chinese Academy of Agricultural Sciences, Dapeng New District, Shenzhen City, Guangdong Province, China. *M. micrantha* seeds were cultured in plastic Petri dishes with wet filter paper under greenhouse conditions (a greenhouse with daily cycles of 16 h/light and 8 h/dark at 25 °C and a relative humidity of approximately 80%) for approximately 1 week until they took root and germinated. The plants were subsequently transplanted into Hoagland liquid medium (KNO_3_: 506 mg/L; NH_4_NO_3_: 80 mg/L; KH_2_PO_4_: 136 mg/L; MgSO_4_: 241 mg/L; FeNaEDTA: 36.7 mg/L; KI: 0.83 mg/L; H_3_BO_3_: 6.2 mg/L; MnSO_4_: 22.3 mg/L; Na_2_MoO_4_: 0.25 mg/L; ZnSO_4_: 8.6 mg/L; CuSO_4_: 0.025 mg/L; CoCl_2_: 0.025 mg/L; Ca(NO_3_)_2_: 945 mg/L; pH = 5.8) and grown in a greenhouse under constant illumination until the fourth leaf stage.

### 4.2. Identification of the M. micrantha MP Gene

The amino acid sequence of the *MONOPTEROS* (*MP*) (Gene ID: AT1G19850) gene in *A. thaliana* was downloaded from NCBI. We subsequently used the protein sequence of *MP* in *A. thaliana* to align to the protein sequences of four plants (*M. micrantha*, *Chrysanthemum nankingense*, *Helianthus annuus*, and *Lactuca sativa*; the species genome information is shown in Supplementary Table S1 via BLASTP with an E-value < 10^−20^. Candidate *MP* genes from these four plants were used to construct a phylogenetic tree via the neighbor–joining method in MEGA (version 11.0.13) with parameters of P-distance modeling, partial deletion of gaps, and 1000 bootstrap replicates [57,58,59]. Multiple sequence alignments of amino acid sequences were performed via ClustalW (version 2.1).

The transcriptome data for different tissues of *M. micrantha*, including flowers, leaves, roots, stems, and stem apices, were downloaded from NCBI (SRR8857616-SRR8857640) and used for subsequent gene expression analysis. To eliminate errors generated in sequencing, unreliable reads were filtered according to the following standards: (1) a 50% base Phred quality score was less than 20%; and (2) a read contained more than 10% ambiguous residues (‘N’). After filtering, the clean reads were mapped to the reference genome of *M. micrantha* via HISAT2 v2.1.0 with the default parameters [60]. Read counts were calculated with HTSeq-count v2.1.0 [61] via the results from SAMtools v1.1.3 [62], and transcripts per million (TPM) values were then calculated for every gene in the samples. GraphPad Prism 8.4.3 (Home-GraphPad) was used to map the expression of the *M. micrantha* candidate genes in different tissues.

### 4.3. RNA Extraction and Gene Cloning

Total RNA was extracted from *M. micrantha* leaves via a Plant Total RNA Isolation Kit Plus (FOREGENE, Chengdu, China) following the manufacturer’s protocols. The first strand of complementary DNA (cDNA) was synthesized from 1 μg of total RNA via the Hifair^®^ III 1st Strand cDNA Synthesis Kit (Yeasen Biotech, Shanghai, China). For amplification of the cDNA sequence of *MmMP*, PCR primers were designed via Primer Premier 5 (Supplementary Table S2). The amplification procedure was 94 °C for 5 min, followed by 35 cycles of 94 °C for 10 s, 55 °C for 20 s, and 10 s at 72 °C, with a final elongation for 7 min at 72 °C. The expected product was cloned and inserted into the pMD18-T vector (TaKaRa, Dalian, China) and transformed into *E. coli* DH5α-competent cells (Yeasen Biotech, Shanghai, China). The plasmid was extracted and identified via sequencing.

### 4.4. Synthesis of dsRNA

A plasmid was used as a template to amplify dsRNA target gene sequences. The T7 RNA polymerase promoter (TAATACGACTCACTATAGGG) was added to the 5′ end of the *dsMmMP* primer (Supplementary Table S2). The amplified PCR products were purified and used as DNA templates, and dsRNA was synthesized using a T7 RNAi Transcription Kit (Vazyme, Nanjing, China). In addition, the dsRNA of enhanced green fluorescent protein (EGFP), a nonendogenous gene, was synthesized and used as a negative control (Supplementary Table S2). The synthesized dsRNA was collected via ethanol precipitation. Purified dsRNA was examined by agarose gel electrophoresis to determine its integrity and then stored at −20 °C before use.

### 4.5. Delivery of dsRNA and Phenotypic Analysis

dsRNA was applied to the roots of *M. micrantha* by soaking. The dsRNA application method was as follows: plant roots were immersed in 2.5 mL glass bottles containing 0.3 μg/μL dsRNA solution, and the treated plants were cultured at 25 °C and 80% relative humidity. The experiment was divided into a treatment group (*dsMmMP*) and two control groups (CK, water control; *dsEGFP*, negative control). Add 0.3 μg/μL dsRNA on the first day of treatment and not continuing to add it later. To determine RNAi efficiency, total RNA was extracted from the roots of plants treated with *dsMmMP*, *dsEGFP*, and CK on the first, third, fifth, and seventh days. The expression of *MmMp* gene in these days was detected. In addition, to determine the plant phenotypic effect, one plant was taken as a biological replicate, and each treatment had at least eight biological replicates. In the phenotypic measurement experiment, we added 0.3 μg/μL dsRNA every other day; the length of the longest adventitious root and the plant height were measured at 3 d, 5 d, 7 d, 10 d, 13 d, and 15 d after treatment. The number of adventitious roots and the fresh and dry root weights of all adventitious roots were measured at 15 d after treatment.

### 4.6. Gene Expression Analysis by Quantitative Real-Time PCR (qRT–PCR)

To reduce DNA contamination, a 5 × gDNA Digester Mix (Hifair^®^ III 1st Strand cDNA Synthesis Kit) was used to remove gDNA contamination from the reverse transcription products. qPCR was performed via a CFX96 real-time PCR system (Bio-Rad, Hercules, CA, USA) with three biological replicates. Two internal controls, *GAPDH* (AGI: AT1G13440) and *UBQ10* (AGI: AT4G05320), were selected from previous studies [63] as relevant reference genes for qRT–PCR in *M. micrantha*. The gene expression levels were calculated via the 2^−ΔΔCt^ method [64]. The sequences of the gene-specific primers used for qPCR are listed in Supplementary Table S2.

### 4.7. Statistical Analysis

The experimental data were calculated as the mean ± standard error (SE), and one-way ANOVA was used to determine significant differences in plant-silencing efficiency. Tukey’s multiple comparisons and independent samples *t*-tests were performed in this study (*p* < 0.05), and the statistical analyses were performed in IBM SPSS Statistics version 29.0.1.0.

### 4.8. Safety Assessment of DsMmMP

Rice and tomato seeds were cultured in plastic Petri dishes with wet filter paper under greenhouse conditions (a greenhouse with daily cycles of 16 h/light and 8 h/dark at 25 °C and a relative humidity of approximately 80%) for approximately 1 week until they took root and germinated. The plants were subsequently transplanted into Hoagland liquid medium and grown in a light-constant greenhouse.

In accordance with previous studies, the MP genes of rice (*OsARF11*, GenBank: LOC4337309; Sims et al., 2021) [65] and tomato (*SlARF5*, GenBank: HM195248.1; Wu et al., 2011) [66] were downloaded from the National Center for Biotechnology Information (NCBI) database. The amino acid sequence alignments were performed via DNAMAN software (version 8; Lynnon Corporation, San Ramon, CA, USA). The alignments of the *dsMmMP* binding sequence of *M. micrantha* and the MP gene of rice or tomato were also performed via DNAMAN software.

*DsMmMP* was applied to the roots of rice and tomato plants by soaking. The dsRNA application method was the same as that described previously. After 3 d, the MP gene expression in the root tissues of rice (*OsARF11*) and tomato (*SlARF5*) plants was determined via qRT–PCR (Supplementary Table S2). The gene expression levels were calculated via the 2^−ΔΔCt^ method [63,64]. The morphological phenotypes, including plant height, root number, root length, and root fresh weight, were measured after *dsMmMP* treatment for 15 d. This phenotypic analysis was conducted with at least eight biological replicates for each treatment.

## 5. Conclusions

In this study, an RNA interference system was successfully constructed for the invasive weed *M. micrantha*. We identified a gene encoding the *MmMP* auxin response protein of *M. micrantha* and verified the function of this gene, which is involved in root growth and development, via RNAi-mediated technology. These results suggest that *MmMP* plays an important role in root development in *M. micrantha* and therefore provides a potential target for the development of RNAi-based herbicides that might contribute to the biological control of invasive weeds.

## Data Availability

Data is contained within the article and Supplementary Materials.

## Supplementary Materials

The supporting information can be downloaded at: https://www.mdpi.com/article/10.3390/ijms252312678/s1.
