# Supplementary material for: Silencing of the MP Gene via dsRNA Affects Root Development and Growth in the Invasive Weed Mikania micrantha
Source: Int J Mol Sci. 2024 Nov 26;25(23):12678. doi: 10.3390/ijms252312678 (PMC11641549; doi:10.3390/ijms252312678)
Supplement: Supplementary file 1 [file ijms-25-12678-s001.zip › Supplementary Figure S1.pdf]

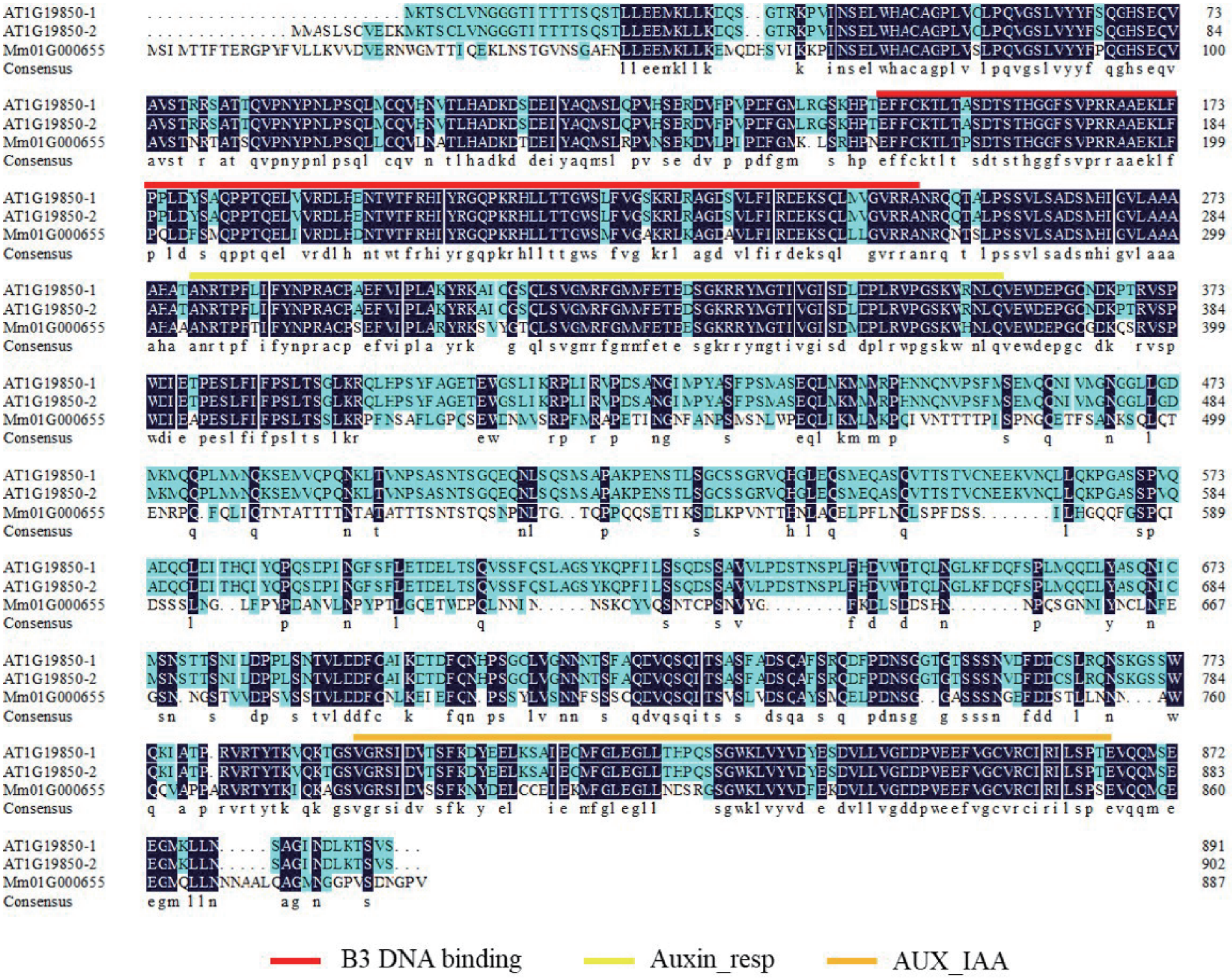

Supplementary Figure S1 Multiple sequence alignment of *MPs* from *M. micrantha* and *A. thaliana* (AT1G19850-1 and AT1G19850-2). The conserved domains of *MPs* with the plant-specific B3-DNA binding domains、Auxin-resp and AUX-IAA are indicated by red, yellow and orange lines, respectively.
