# Supplementary material for: Silencing of the MP Gene via dsRNA Affects Root Development and Growth in the Invasive Weed Mikania micrantha
Source: Int J Mol Sci. 2024 Nov 26;25(23):12678. doi: 10.3390/ijms252312678 (PMC11641549; doi:10.3390/ijms252312678)
Supplement: Supplementary file 1 [file ijms-25-12678-s001.zip › Supplementary Figure S2.pdf]

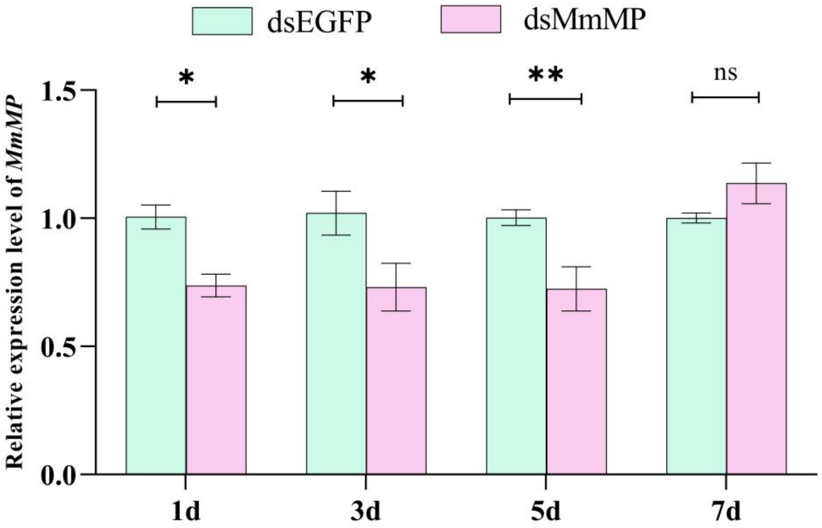

Supplementary Figure S2 The abundance of *MmMP* transcripts was measured at different time points after soaking the roots with *dsEGFP* and *dsMmMP*. Each bar represents the mean ± SD derived from 3 biological replicates. T-test was used to determine significant differences in plant silencing efficiency in this study (* $p < 0.05$; ** $p < 0.01$; *** $p < 0.001$).“ns” Represents no significant difference between *dsEGFP* and *dsMmMP* treatment. *dsEGFP* indicates the dsRNA of the enhanced green fluorescent protein (*EGFP*) and *dsMmMP* indicates double-stranded RNA of the *M. micrantha MmM*P gene.
