# Supplementary material for: Silencing of the MP Gene via dsRNA Affects Root Development and Growth in the Invasive Weed Mikania micrantha
Source: Int J Mol Sci. 2024 Nov 26;25(23):12678. doi: 10.3390/ijms252312678 (PMC11641549; doi:10.3390/ijms252312678)
Supplement: Supplementary file 1 [file ijms-25-12678-s001.zip › Supplementary Figure S3.pdf]

A

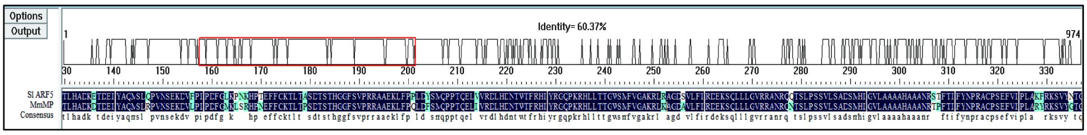

B

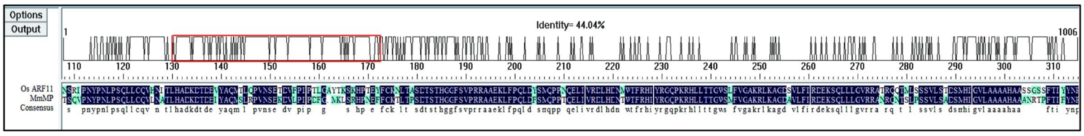

Supplementary Figure S3 Comparison of the amino acid sequences of *M. micrantha*, *O. sativa* and *S. lycopersicum*; A: Comparison between *MmMP* and *SlARF5A*; B: Comparison between *MmMP* and *OsARF11*
