# Supplementary material for: Silencing of the MP Gene via dsRNA Affects Root Development and Growth in the Invasive Weed Mikania micrantha
Source: Int J Mol Sci. 2024 Nov 26;25(23):12678. doi: 10.3390/ijms252312678 (PMC11641549; doi:10.3390/ijms252312678)
Supplement: Supplementary file 1 [file ijms-25-12678-s001.zip › Supplementary Figure S4.pdf]

A

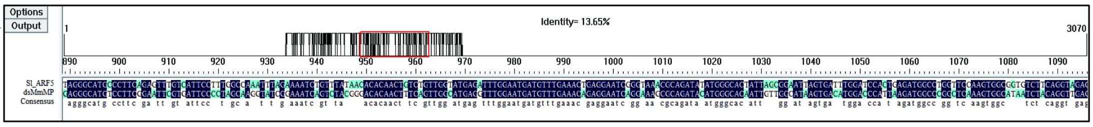

B

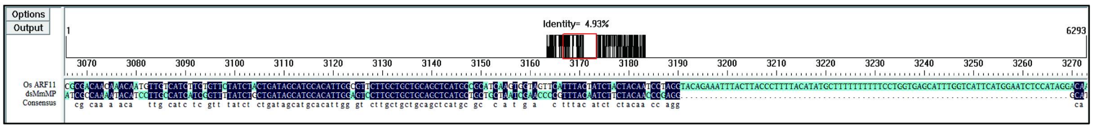

Supplementary Figure S4 Comparison of the RNAi target sequences of *M. micrantha* with the nucleotide sequences of *O. sativa* and *S. lycopersicum*. A: Comparison between *dsMmMP* and *SlARF5A*; B: Comparison between *dsMmMP* and *OsARF11*
