# Supplementary material for: Silencing of the MP Gene via dsRNA Affects Root Development and Growth in the Invasive Weed Mikania micrantha
Source: Int J Mol Sci. 2024 Nov 26;25(23):12678. doi: 10.3390/ijms252312678 (PMC11641549; doi:10.3390/ijms252312678)
Supplement: Supplementary file 1 [file ijms-25-12678-s001.zip › Supplementary Figure S5.pdf]

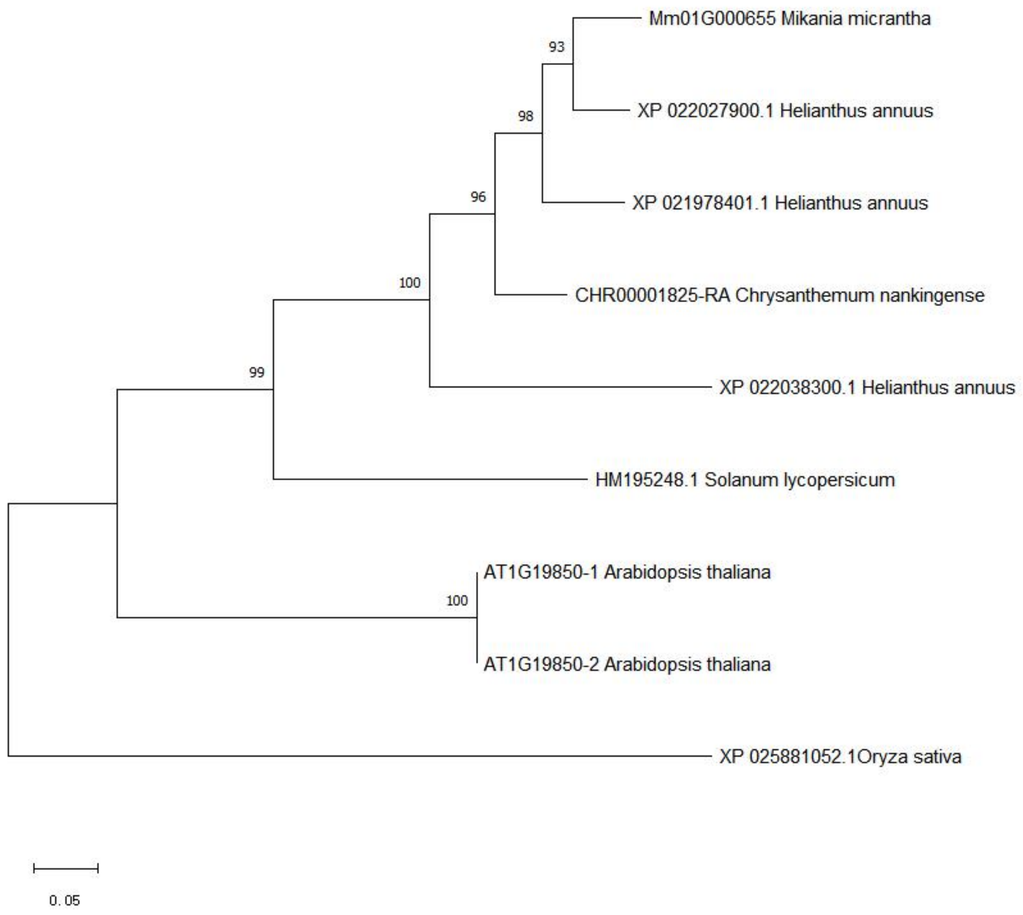

Supplementary Figure S5 Phylogenetic analysis of *MP* genes in *M. micrantha* and other species including *A. thaliana*, *C. nankingense*, *H. annuus*, *S. lycopersicum* and *O. sativa*. Numbers on the branches represent bootstrap values obtained from 1000 replicates. The branch lengths are proportional to the percentage of sequence difference (scale bar, 0.05% difference).
