# Supplementary material for: Silencing of the MP Gene via dsRNA Affects Root Development and Growth in the Invasive Weed Mikania micrantha
Source: Int J Mol Sci. 2024 Nov 26;25(23):12678. doi: 10.3390/ijms252312678 (PMC11641549; doi:10.3390/ijms252312678)
Supplement: Supplementary file 1 [file ijms-25-12678-s001.zip › Supplementary_tables.pdf]

Supplementary Table S1 Genome information for the four species

Species name	Genome download information
Mikania micrantha	GCA_009363875.1
Chrysanthemum nankingense	http://www.amwayabrc.com/download.htm
Helianthus annuus	GCA_002127325.2
Lactuca sativa	GCA_002870075.4

Supplementary Table S2 Primers for gene cloning、dsRNA synthesis and fluorescence quantitative PCR

Primer name	Forward (5’ to 3’)	Reverse (5’ to 3’)	Purpose
MmMP	GGCTGGAGCATGTTTGTTGG	ACTCGAAGTGAGGGATGGGA	gene clone
ds*MmMP*	TAATACGACTCACTATAGGG<br>GGCTGGAGCATGTTTGTTGG	TAATACGACTCACTATAGGG<br>ACTCGAAGTGAGGGATGGGA	Synthesis of dsRNA
dsEGFP	TAATACGACTCACTATAGGG<br>TGAGCAAGGGCGAGGAG	TAATACGACTCACTATAGGG<br>CGGCGGTCACGAACTCCAG	Synthesis of dsRNA
q*MmMP*	GGGCGTCTTCTAGCAATGGTGAG	GCGAGCAGGAGGAGCAACTTG	qRT-PCR
q*UBQ*	TGCCGACTACAACATCCAGAA	CCGCAGTAATGCCGATCAAA	qRT-PCR
q*GAPDH*	TCCAGGAACCCAGACGAGAT	AGACCCTCAACAATGCCAAA	qRT-PCR
Sd-OsARF11	AGCAGCAGCAGCAGGAGAG	AGTAGATGGCAATGATACGGAGGAG	qRT-PCR
Sd-UBQ	GCCGACTACAACATCCAGAAGG	GCCGACTACAACATCCAGAAGG	qRT-PCR
Sd-GAPDH	CGTCGCATCGCACTTGTAGC	CCAGATCGAGATCGGAGGAAGC	qRT-PCR
Fq- SlARF5	AAGATGCTGATAAGACCTCCTCCTG	TCCTGCTGGCTGTACTACATTCC	qRT-PCR
Fq-UBO	CTTCACTTGGTCCTTCGTCTT	TGTCAGAACTTTCCACCTCAA	qRT-PCR
Fq- GAPDH	TGGGGAGCAAAAGACAGCAATG	ACAGGGTTCCGTTCTTCTCTGG	qRT-PCR

Supplementary Table S3 Selected targeted genes

Functional verification of genes	Functions Available	Genes screened after Blastp comparison		Identity (%)	E-value
AtMP（AGI：	Auxin	*M.*	Mm11G025717	69.18	9.67E-138

AT1G19850）	response factor, MP mutants fail to form roots	*micrantha*	Mm11G025701	69.18	1.27E-137
			Mm12G029767	66.94	3.57E-162
			Mm10G025061	65.47	1.32E-159
			Mm07G018880	64.31	4.39E-158
			Mm01G001674	64.11	2.41E-157
			Mm05G011974	63.24	9.17E-157
			Mm09G023255	62.91	1.49E-147
			Mm01G000655	56.02	0
		H. annuus	XP_021978401.1	76.85	0
			XP_022038300.1	73.33	0
			XP_022027900.1	66.82	2.16E-79
			XP_021981467.1	66.57	7.56E-154
			XP_022027148.1	66.49	1.04E-155
			XP_022035297.1	66.29	7.37E-160
			XP_022027788.1	65.78	1.98E-158
			XP_022012151.1	64.69	1.20E-153
			XP_021993406.1	64.11	7.39E-159
			XP_021989698.1	63.97	1.77E-155
			XP_022009902.1	63.84	4.91E-157
			XP_022016074.1	62.73	1.25E-159
			XP_022012442.1	62.60	2.30E-156
		C. nankingense	CHR00001825-RA	59.15	0
			CHR00072492-RA	67.12	2.73E-162
			CHR00006287-RA	62.21	1.31E-157
			CHR00040805-RA	60.78	1.05E-154
			CHR00043356-RA	61.02	5.41E-135
			CHR00057310-RA	59.66	6.33E-135
		L. sativa	XP_023772550.1	73.12	0
			XP_023757391.1	72.25	1.38E-66
			XP_023732183.1	70.14	1.09E-157
			XP_023739623.1	67.88	1.10E-157
			XP_023770043.1	67.70	2.00E-156
			XP_023733022.1	66.01	5.70E-158
			XP_023767431.1	63.84	4.10E-156
			XP_023747631.1	62.05	3.28E-159
			XP_023734807.1	60.18	0

Supplementary Table S4 Selected targeted gene sequences

Name of gene	Target gene sequence
Mm01G000655	ATGAGTATTATGACCACTTTCACAGAAAGAGGGCCTTATTTTGTTCTTCTT<br>AAAGTTGTTGATGTAGAAAGGAATTGGGGTATGACCACCATTCAAGAGAAGCT<br>AAATTCAACAGGTGTGAACAGTGGGGCACACAACTTACTTGAGGAGATGAAAC<br>TGTTGAAGGAAATGCAAGACCATTCTGTCATAAAGAAGCCAATAAATTCTGAG<br>CTATGGCATGCTTGTGCTGGCCCTTTGGTGAGCCTTCCACAGGTTGGAAGCCTT<br>GTTTATTACTTCCCACAAGGACATAGCGAACAGGTTGCGGTCTCGACAAATAG<br>AACAGCAACATCACAAGTTCCTAATTATCCAAATCTTCCATCTCAGCTGTTGTG<br>CCAAGTTTTAAATGCTACCTTACATGCAGATAAAGACACCGATGAGATCTATGC<br>CCAAATGAGCCTTCGACCCGTGAATTCGGAAAAAGATGTCCTTCCTATACCAG<br>ACTTTGGGATGAAATTAAGTAGACATCCAAATGAGTTCTTTTGCAAGACTTTGA<br>CCCCGAGCGATACAAGCACTCATGGCGGCTTTTCTGTACCACGTAGAGCTGCA<br>GAAAAACTTTTTCCGCAGTTGGATTTTTCAATGCAGCCTCCTACTCAAGAGCTT<br>ATTGTCCGAGATTTACATGATAACACGTGGACATTCCGTCATATATACCGCGGG<br>CAGCCAAAACGACACCTACTTACAACGGGCTGGAGCATGTTTGTTGGTGCTAA<br>AAGGCTTAAAGCTGGTGATGCAGTTCTGTTTATCAGGGATGAGAAGTCACAGT<br>TATTATTGGGAGTTCGGCGTGCTAATCGCCAAAATACATCCTTGCCATCATCGG<br>TTTTATCTGCTGATAGCATGCACATTGGAGTCCTTGCTGCTGCAGCTCATGCTG<br>CTGCTAATCGAACCCCGTTTACAATCTTCTACAACCCGAGGGCATGTCCTTCGG<br>AATTCGTGATTCCCCTAGCAAGGTATCGGAAATCAGTCTACGGGACACAACTTT<br>CAGTTGGCATGAGGTTTGGAATGATGTTTGAAACAGAGGAATCAGGAAAGCGC<br>AGATACATGGGCACAATTGTTGGCATAAGTGACATGGACCCATTAAGATGGCC<br>CGGCTCAAAGTGGCATAATCTACAGGTTGAGTGGGACGAGCCTGGTTGTGGTG<br>ACAAGCAAAGTAGAGTGAGCCCATGGGACATTGAGGCCCCTGAAAGCCTCTTC<br>ATTTTCCCATCCCTCACTTCGAGTCTTAAACGACCCTTTAATTCAGCTTTTCTGG<br>GACCACAATCCGAATGGGACAATATGGTAAGCCGTCCATTCATGCGGGCCCCG<br>GAAACCATAAATGGAAACTTTGCAAACCCTTCAATGTCTAACTTATGGCCAGA<br>ACAACTGATCAAAATGTTGATGAAACCTCAAATTGTTAACACCACCACAACGC<br>CCATTTCACCCAATGGTCAAGAGACTTTTTCTGCCAACAAAAGCCAGTTACAAA<br>CCGAAAACCGCCCTCAGTTTCAACTTATTCAAACCAACACCGCCACAACCACC<br>ACCAACACCGCCACTGCAACCACAACGTCAAACACATCAACGCAATCCAACCC<br>AAATCTTACTGGAACCCAACCACCCCAACAATCAGAAACCATAAAATCTGACC<br>TGAAACCAGTCAACACTACACATAATCTTGCACAAGAGCTTCCGTTTCTTAACC<br>AGTTATCTCCATTTGATTCAAGCATACTCCACGGTCAACAATTTGGGTCTCCTC<br>AGATTGATTCATCCAGCCTTAACGGGCTATTCCCGTATCCTGATGCTAATGTAC<br>TGAACCCGTATCCGACTCTTGGGCAGGAAACATGGGACCCACAGTTGAATAAC<br>ATTAACAACTCCAAATGTTATGTACAATCAAACACATGCCCCTCAAACGTATAT<br>GGTTTCAAAGACTTGTCGGATGATAGCCACAATAATCCCCAGAGTGGCAACAA<br>CATATACAACTGTCTCAACTTTGAAGGTAGCAATAATGGTAGCACAGTTGTCGA<br>TCCTTCGGTTTCAAGTACTGTTTTGGATGATTTCTGCAACTTGAAAGAGATCGA<br>GTTTCAAAACCCGTCTAGTTATTTAGTTAGTAACAATTTCAGCTCAAGCTGCCA<br>AGATGTTCAATCACAAATTACTTCAGTTAGCCTTGTGGATTCTCAAGCTTATTCT

	ATGCAAGAACTGCCTGATAACTCGGGTGGGGCGTCTTCTAGCAATGGTGAGTTT<br>GATGATAGCACACTTTTGAATAACAACGCATGGCAACAAGTTGCTCCTCCTGCT<br>CGCGTACGAACTTACACGAAGATTCAAAAGGCGGGATCCGTTGGGAGGTCTAT<br>AGATGTGTCAAGTTTCAAGAACTACGATGAACTTTGTTGTGAGATTGAGAAGA<br>TGTTTGGACTTGAAGGATTGCTTAATGATTCAAGAGGGTCGGGTTGGAAGTTGG<br>TTTATGTTGATTTTGAGAAGGATGTTCTACTAGTTGGAGATGACCCATGGGAGG<br>AATTCGTCGGGTGTGTTAGATGCATCAGGATTCTGTCACCCTCTGAAGTTCAGC<br>AGATGGGTGAAGAGGGAATGCAGCTACTCAACAACAATGCGGCATTACAGGCC<br>GGGATGAACGGTGGGCCAGTATCAGACAACGGGCCAGTGTTATAA

Supplementary Table S5 TPM value of *MmMP*

	Sample 1	Sample 2	Sample 3	Sample 4	Sample 5	Mean ± SE
Flower	64.7	29.9	47.9	34.4	18.7	39.1 ± 7.9
Leaf	4.9	3.8	6.2	0.8	4.4	4.0 ± 0.9
Root	33.2	26.6	25.2	36.3	37.2	31.7 ± 2.5
Stem	9.8	11.7	12.4	9.9	9.1	10.6 ± 0.6
StemApex	45.7	33.6	48.5	43.0	41.3	42.4 ± 2.5
